# Effect of preoperative nutritional risk index on 30-day postoperative complications in patients with gastric cancer: a retrospective cohort study

**DOI:** 10.3389/fonc.2025.1475381

**Published:** 2025-06-16

**Authors:** Yingfeng Zou, Ling Li, Kui Jia, Lei Tian, Minying He, Debin Huang

**Affiliations:** 1 Department of Gastrointestinal Surgery, The First Affiliated Hospital of Guangxi Medical University, Nanning, China; 2 Department of Nursing, The First Affiliated Hospital of Guangxi Medical University, Nanning, China

**Keywords:** gastric cancer, nutritional risk index, postoperative complication, surgery, cancer

## Abstract

**Background:**

Preoperative nutritional status in patients with gastric cancer after surgery has attracted considerable interest. The nutritional risk index (NRI) has been widely used as a convenient and effective nutritional assessment index, but the relationship between preoperative NRI and postoperative complications in patients with gastric cancer has not been adequately studied. Our study aimed to investigate the effects of preoperative NRI on 30-day postoperative complications in patients with gastric cancer.

**Methods:**

This retrospective analysis investigated 578 patients with gastric cancer. Preoperative NRI calculations were based on serum albumin levels and body weight, and receiver operating characteristic curves were used in analyzing NRI values and establishing optimal cutoff points. Patients were categorized into two groups according to cutoff value: low NRI group (NRI<96.7) and high NRI group (NRI≥96.7). The hazard ratio (HR) for postoperative complications was calculated through Cox regression analysis and adjusted for potential confounders, and the effects of NRI on postoperative complications in patients with gastric cancer were examined. In addition, we conducted subgroup analyses to examine whether there was an interaction between the effect of NRI on the cumulative incidence of postoperative complications and other confounding factors.

**Results:**

Of the 578 patients with gastric cancer who underwent radical surgery, 120 (20.8%) experienced postoperative complications. The optimal NRI threshold of 96.7 was identified using ROC curve analysis. Cox regression analysis demonstrated that preoperative NRI was independently associated with 30-day postoperative complications after adjusting for confounding factors (HR=0.93; 95%CI: 0.90–0.96; *P*<0.001). Patients in the low NRI group had significantly higher rates of postoperative complications than those in the high NRI group(HR=2.89, 95%CI:1.71–4.88; *P*<0.001). The cumulative incidence analysis revealed a higher risk of postoperative complications over time in the low NRI group compared with the high NRI group (*P*<0.001). These associations remained robust in subgroup analyses.

**Conclusions:**

NRI is an independent predictor of 30-day postoperative complications in gastric cancer patients and is a convenient and useful nutritional screening tool for identifying patients with gastric cancer at high risk of postoperative complications.

## Introduction

Gastric cancer, a prevalent malignancy in oncology, poses a considerable global health threat and has an annual incidence exceeding one million worldwide, resulting in approximately 770,000 deaths (1). The incidence and mortality rates of gastric cancer are gradually increasing globally, posing formidable challenges to the diagnosis and treatment of the disease. Currently, surgical treatment is considered the primary therapeutic approach (2). However, early postoperative prognosis remains concerning because of the high incidence of complications, which considerably affect patients’ recovery and quality of life (3).

Postoperative complications are among the adverse prognostic factors in cancer care. Their occurrence prolongs hospitalization duration, increases economic burden, and leads to unfavorable prognoses (4, 5). The incidence of postoperative complications in patients with gastric cancer can be as high as 40%, and early complications appear within 30 days after surgery (6, 7). These complications may lead to systemic inflammation, potentially reducing immune response in patients with cancer (8). Therefore, effectively identifying high-risk patients prone to postoperative complications before undergoing surgery for gastric cancer is crucial.

Malnutrition potentially affects as many as 40%–80% of patients with gastric cancer, which along with weakened immunity can increase the likelihood of postoperative complications (9, 10). Thus, the preoperative assessment of the nutritional status of these patients is crucial. The nutritional risk index (NRI), designed and implemented by the Veterans Affairs Total Parenteral Nutrition Cooperative Study Group, is a nutritional assessment tool determined by serum albumin and body weight (11). The NRI enables the routine and straightforward assessment of patients’ nutritional status before surgery and can aid in predicting postoperative complications and outcomes in patients with cancer. The NRI is extensively utilized for patients with cardiac surgery (12)and breast cancer (13), but its applicability to patients with gastric cancer has not been well established. This study seeks to examine the influence of preoperative NRI on postoperative complications within 30 days for patients with gastric cancer, aiming to assess NRI values during nutritional screening for preoperative patients with gastric cancer.

## Methods

### Data source and study population

This study retrospectively included patients who underwent radical gastrectomy for gastric cancer in the Department of Gastrointestinal Surgery at the First Affiliated Hospital of Guangxi Medical University from January 2019 to June 2023. All patient data were followed up by dedicated personnel for 30 days after the surgery. The inclusion criteria were as follows: (1) age ≥ 18 years; (2) confirmed diagnosis of gastric cancer and initial curative surgery; and (3) comprehensive medical history. The exclusion criteria were (1) preoperative adjuvant chemotherapy or radiotherapy; (2) concurrent primary tumors; (3) cases requiring emergency surgery because of gastric perforation and bleeding; and (4) cases undergoing concomitant resection of other organs during surgery. A total of 578 patients were included in this study, which employed a retrospective research design and obtained a waiver for informed consent. The study was approved by the ethics committee of the First Affiliated Hospital of Guangxi Medical University (2023-k293-01).

### Data collection and definitions

We collected several variables, including (1) general information: gender, age, BMI, smoking history, drinking history, comorbidities (hypertension, diabetes, coronary heart disease, and cerebral infarction); (2) laboratory parameters: albumin, hemoglobin, prealbumin, C-reactive protein, WBC, CA199, and AFP; (3) data related to surgical and tumor characteristics: gastrectomy, surgical approach, ASA class, TNM staging, tumor differentiation, intraoperative blood transfusion, operation time, intraoperative blood loss, preoperative nutritional intervention; (4) outcome variables: complications within 30 days after surgery. Use the following formula to calculate the NRI (11): NRI = 1.519 × albumin (g/L) + [41.7 × (weight/Wlo)]. Ideal weight (WLo) is calculated from height (H) and gender with the Lorenz formula (14). For men, Wlo (kg)=H (cm)−100−[(H−150)/4]. For women, Wlo (kg)=H(cm)–100−[(H−150)/2.5]. When the actual weight is greater than the ideal weight, the actual weight/ideal weight is taken as 1. The study population was divided into two groups according to the optimal cutoff value for the results of the ROC analysis: the low NRI group(NRI<96.7)and the high NRI group(NRI≥96.7). The Clavien–Dindo Classification system was used in categorizing postoperative complications into grades I, II, III, IV, and V (15). The presence of more than one complication in a patient was recorded as the highest level of complication. Frequent postoperative complications in patients with gastric cancer included pulmonary infection, gastrointestinal fistula, gastric atony, pleural effusion and others.

### Statistical analysis

Continuous variables were presented as either mean ± standard deviation or median (interquartile range), whereas categorical variables were depicted as numbers and percentages (%). Intergroup comparisons were performed using t-tests, chi-square tests, and Fisher’s exact tests. The receiver operating characteristic (ROC) curve was used in determining the optimal cutoff value for NRI. Model 1 remained unadjusted, Model 2 was adjusted for age, sex, and BMI, Model 3 included adjustments for the variables in Model 2 and gastrectomy, surgical approach, intraoperative blood transfusion, operation time, and intraoperative blood loss, and Model 4 included adjustments for the variables in Model 3 and preoperative nutritional intervention. Subgroup analysis was conducted to confirm the reliability of the results, and stratification was based on gender, age (<60 and ≥60 years), BMI, gastrectomy, surgical approach, TNM staging, intraoperative blood transfusion and preoperative nutritional intervention. The cumulative incidence plot was employed, and a log-rank test was used to assess the difference in the cumulative risk of postoperative complications within 30 days between the low- and high-NRI groups. We excluded variables with missing values exceeding 20%, and for continuous variables with less than 5% missing values, the mean or median was used for replacement. All studies analyzed data using the statistical software packages R (http://www.R-project.org, R Foundation) and Free Statistics software version 1.8. For all analyses, a two-tailed *P*<0.05 was considered statistically significant.

## Results

### Baseline characteristics of patients

**Figure 1:**
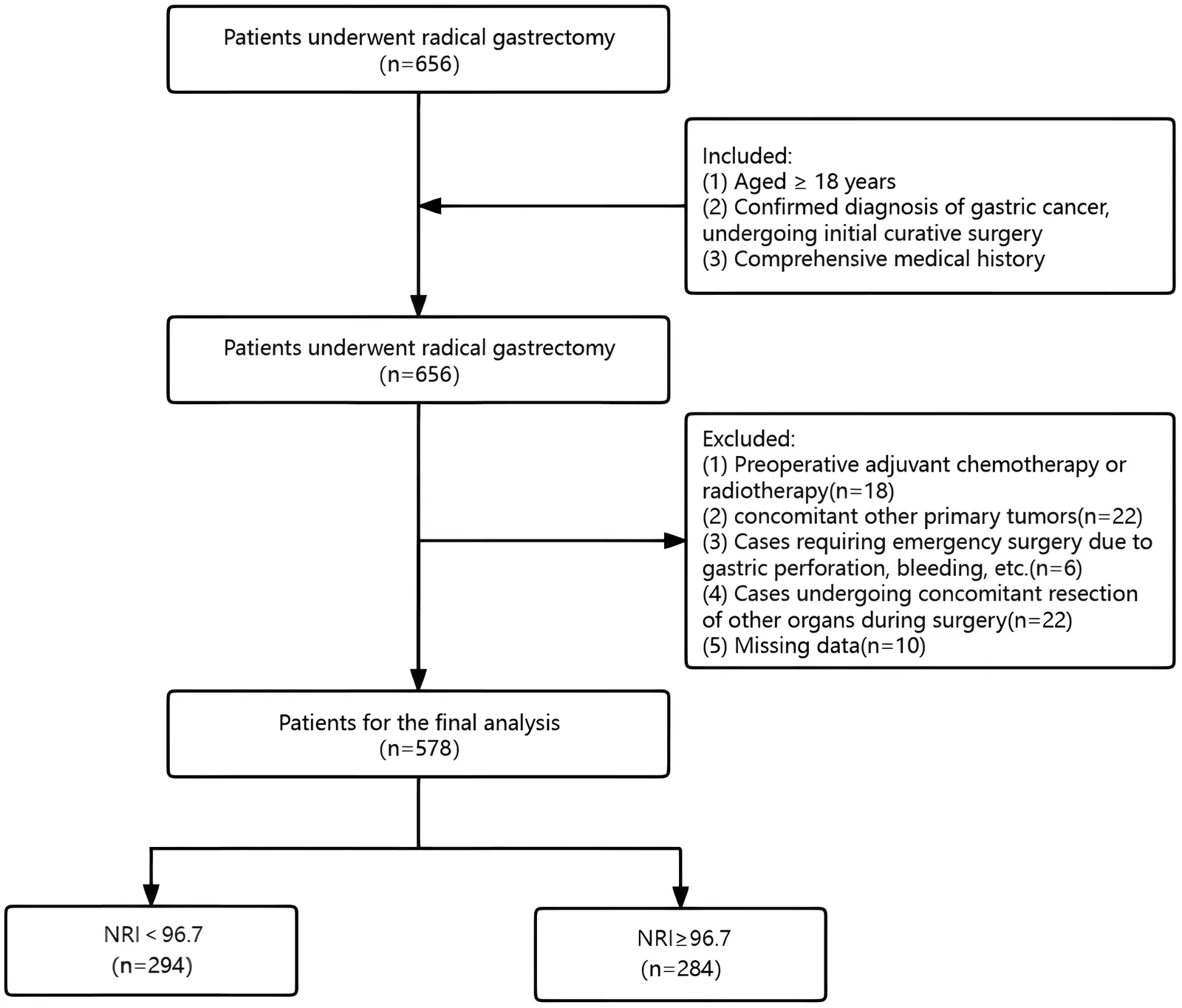
Flowchart of the study population. NRI, nutritional risk index.

A total of 656 patients underwent gastric cancer surgery between January 2019 and June 2023, and 578 eligible patients were finally enrolled for analysis after the exclusion of patients with other surgical procedures and missing key data (**Figure 1**). The optimal cutoff value for NRI was 96.7 according to ROC curve analysis, and the study population was categorized into low-NRI (<96.7) and high-NRI (≥96.7) groups (**Supplementary Figure 1**).

The baseline characteristics of the patients are summarized in **Table 1**. The median age was 60.0 (52.0, 64.0), and 361 (62.5%) were male. A total of 120 (20.8%) patients suffered from postoperative complications. There were 84 (70%) pulmonary infections, 10 (8.3%) GI fistulas, 4 (3.3%) gastroparesis, 4 (3.3%) were pleural effusions, and 18 (15%) other cases in **Supplementary Table 1**. The two groups were compared in terms of age, preoperative nutritional intervention, BMI, smoking, drinking, TNM staging, tumor differentiation, intraoperative blood transfusion, albumin, hemoglobin, prealbumin, C-reactive protein, and WBC. The differences were all statistically significant (*P*<0.05). In addition, patients in the low-NRI group presented a higher incidence of postoperative complications than those in the high-NRI group (28.2% vs. 13%), including grades II (22.4% vs. 7.7%),grade III (4.8% vs. 4.2%) and grade IV (1.0% vs. 1.1%). Additionally, the baseline characteristics of patients with and without complications are presented in **Supplementary Table 2**. Patients with complications had lower NRI values and were more likely to be male compared with patients without complications. Patients with complications had a higher chance of intraoperative blood transfusion and blood loss and required longer operative times than those without.

**Table 1:** Baseline characteristics of the study population according to NRI.

Variables	Total (n=578)	NRI<96.7 (n=294)	NRI≥96.7 (n=284)	*P* value
Age, Median (IQR)	60.0 (52.0, 64.0)	61.0 (55.0, 65.0)	58.0 (49.0, 62.0)	<0.001
Male, n (%)	361 (62.5)	188 (63.9)	173 (60.9)	0.452
BMI, Median (IQR)	21.8 (20.0, 23.8)	20.7 (19.0, 22.5)	22.9 (21.5, 24.6)	<0.001
Smoking,n (%)	217 (37.5)	124 (42.2)	93 (32.7)	0.019
Drinking, n (%)	210 (36.3)	120 (40.8)	90 (31.7)	0.023
Preoperative nutritional intervention, n(%)				<0.001
No	140 (24.2)	40 (13.6)	100 (35.2)	
Yes	438 (75.8)	254 (86.4)	184 (64.8)	
Gastrectomy, n(%)				0.327
proximal	15 ( 2.6)	10 (3.4)	5 (1.8)	
distal	496 (85.8)	247 (84.0)	249 (87.7)	
Total	67 (11.6)	37 (12.6)	30 (10.6)	
Surgical approach,n(%)				0.228
Open	105 (18.2)	59 (20.1)	46 (16.2)	
Laparoscopic	473 (81.8)	235 (79.9)	238 (83.8)	
ASA Class, n(%)				0.380
I	14 ( 2.4)	8 (2.7)	6 (2.1)	
II	446 (77.2)	228 (77.6)	218 (76.8)	
III	112 (19.4)	53 (18.0)	59 (20.8)	
IV	6 ( 1.0)	5 (1.7)	1 (0.4)	
TNM staging, n(%)				<0.001
I	198 (34.3)	74 (25.2)	124 (43.7)	
II	135 (23.4)	83 (28.2)	52 (18.3)	
III	245 (42.4)	137 (46.6)	108 (38.0)	
Tumor differentiation, n(%)				<0.001
Un-classified	3 ( 0.5)	3 (1.0)	0 (0.0)	
Well	434 (75.1)	212 (72.1)	222 (78.2)	
Moderate	116 (20.1)	73 (24.8)	43 (15.1)	
Poor	25 ( 4.3)	6 (2.0)	19 (6.7)	
Hypertension, n (%)	115 (19.9)	55 (18.7)	60 (21.1)	0.466
Diabetes, n (%)	12 ( 2.1)	5 (1.7)	7 (2.5)	0.520
Coronary heart disease, n (%)	5 ( 0.9)	3 (1.0)	2 (0.7)	1.000
Cerebral Infarction, n (%)	5 ( 0.9)	5 (1.7)	0 (0.0)	0.062
Intraoperative blood transfusion	129 (22.3)	89 (30.3)	40 (14.1)	<0.001
Albumin, g/L	37.4 (34.8, 40.1)	34.9 (32.4, 36.4)	40.0 (38.2, 41.8)	<0.001
Hemoglobin, g/L	124.0(108.7, 134.0)	116.0(94.8, 129.0)	127.5(118.7, 139.3)	<0.001
Prealbumin, mg/L	215.3(177.6, 251.8)	194.1(159.1, 223.6)	238.4(206.5, 275.6)	<0.001
C-Reactive Protein, mg/L	8.0 (8.0, 32.3)	8.0 (8.0, 32.3)	8.0 (8.0, 32.3)	0.006
WBC, 109/L	6.1 (5.0, 7.1)	5.9 (4.8, 7.0)	6.2 (5.3, 7.3)	0.008
CA199, U/ml	6.3 (2.5, 17.2)	5.9 (2.2, 18.9)	6.6 (2.9, 16.0)	0.732
AFP, ng/ml	2.2 (1.4, 3.7)	2.3 (1.4, 4.0)	2.1 (1.4, 3.3)	0.149
Operation time, h	4.9 (4.2, 5.6)	4.9 (4.2, 5.6)	4.9 (4.2, 5.7)	0.665
Intraoperative blood loss, ml	100(80, 200)	100(50, 200)	100(100, 200)	0.414
Complication, n (%)				<0.001
No	458 (79.2)	211 (71.8)	247 (87.0)	
Yes	120 (20.8)	83 (28.2)	37 (13.0)	
Grade II	88 (15.2)	66 (22.4)	22 (7.7)	
Grade III	26 ( 4.5)	14 (4.8)	12 (4.2)	
Grade IV	6 ( 1.0)	3 (1.0)	3 (1.1)	
Grade V	0 ( 0.0)	0 (0.0)	0 (0.0)	

NRI, nutritional risk index; BMI, body mass index; ASA, American Society of Anesthesiologists; TNM, Tumor-Node-Metastasis; WBC, white blood cell; AFP, Alpha-Fetoprotein.

### Association between NRI and 30-day postoperative complications

Univariate analysis of the factors affecting 30-day postoperative complications is displayed in **Supplementary Table 3**. Gender, age, NRI, albumin, hemoglobin, prealbumin, gastrectomy, preoperative nutritional intervention, intraoperative blood loss, operation time, surgical approach, and intraoperative blood transfusion may be associated with postoperative complications(*P*<0.05).

The results of the multivariate Cox regression analyses are presented in **Table 2**, including unadjusted model 1, partially adjusted model 2, partially adjusted model 3,and fully adjusted model 4. In fully adjusted model 4, NRI was treated as a continuous variable, and a negative correlation was found between NRI and the incidence of 30-day postoperative complications. Specifically, for every 1 unit increase in NRI, a 7% decrease in the risk of 30-day postoperative complications was found (HR=0.93, 95% CI:0.90–0.96, *P*<0.001). This negative association was maintained in unadjusted model 1, partially adjusted model 2, partially adjusted model 3,and fully adjusted model4. When NRI was used as a categorical variable in the uncorrected model 1, the risk of complications at 30 days postoperatively was higher in the low NRI group compared with the high NRI group (HR=2.63, 95% CI: 1.71–4.03; *P*<0.001), and this association remained significant even after full adjustments for variables (HR=2.89, 95% CI: 1.71–4.88; *P*<0.001).

**Table 2:** Multivariable Cox regression to assess the association of NRI with 30-day postoperative complications.

Variable	Model 1		Model 2		Model 3		Model 4	
	HR(95%CI)	*P* value	HR(95%CI)	*P* value	HR(95%CI)	*P* value	HR(95%CI)	*P* value
NRI	0.94 (0.92~0.97)	<0.001	0.93 (0.90~0.96)	<0.001	0.93 (0.89~0.96)	<0.001	0.93 (0.90~0.96)	<0.001
NRI≥96.7	1(Ref)		1(Ref)		1(Ref)		1(Ref)	
NRI<96.7	2.63 (1.71∼4.03)	<0.001	2.85 (1.76∼4.63)	<0.001	2.96 (1.76∼4.98)	<0.001	2.89 (1.71∼4.88)	<0.001

Model 1: Non-adjusted model.

Model 2: Adjust for age, gender, and BMI.

Model 3: Model 2 + Gastrectomy, Surgical approach, Intraoperative blood transfusion, Operation time, Intraoperative blood loss.

Model 4: Model 3 + Preoperative nutritional intervention.

### Subgroup analysis

**Figure 2:**
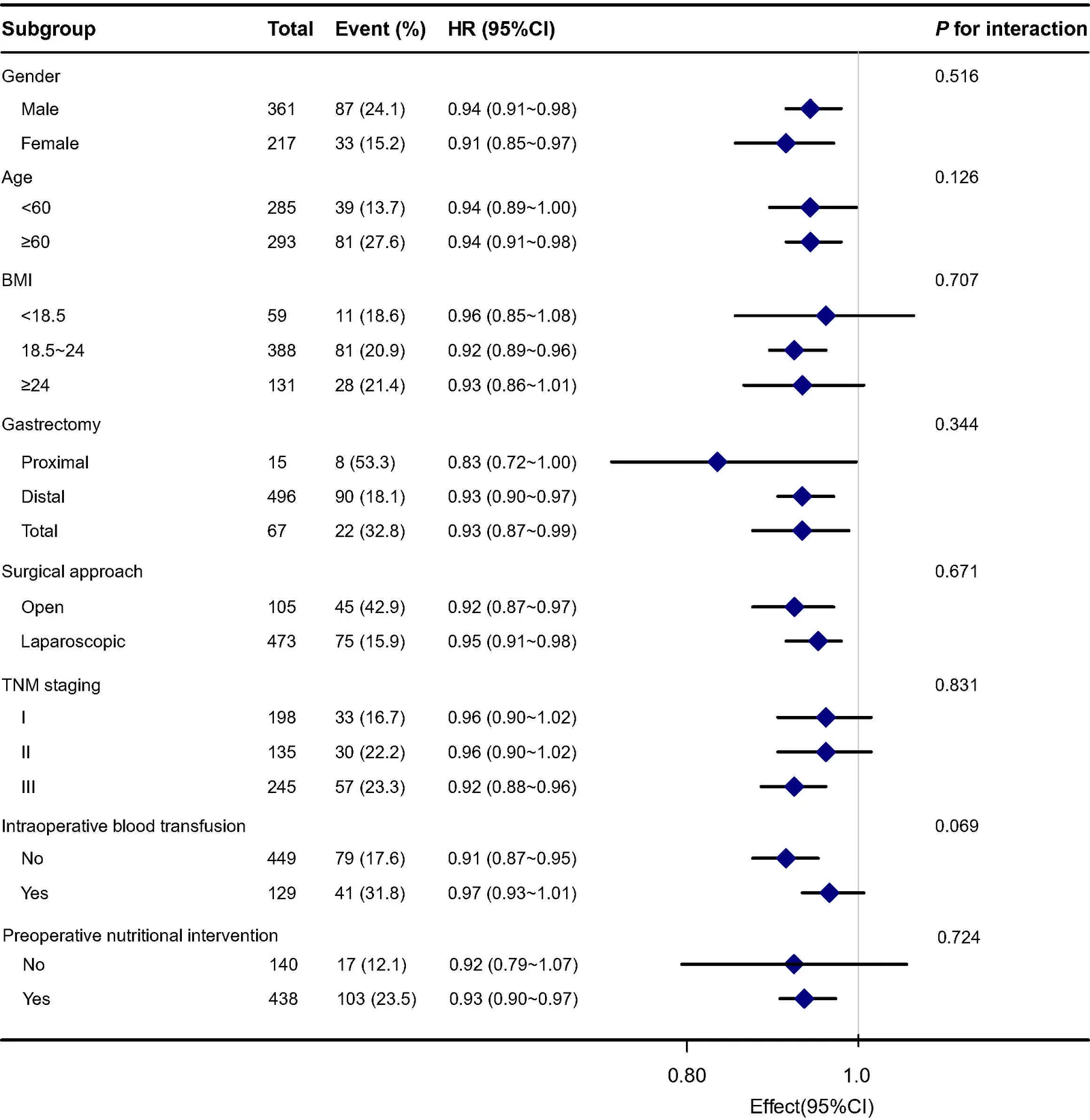
Subgroup analysis for the association between preoperative NRI and 30-day postoperative complications.

We further performed subgroup analysis to test whether the association between NRI and 30-day postoperative complications was stable across subgroups. As shown in **Figure 2**, no significant interactions were observed in the subgroups in terms of gender, age, BMI, gastrectomy, surgical approach, TNM staging, intraoperative blood transfusion and preoperative nutritional intervention after adjusting for confounders (*P* for interaction > 0.05). This result indicates that the results of the negative association between NRI and the risk of 30-day postoperative complications in patients with gastric cancer remain robust regardless of baseline patient characteristics.

### Cumulative incidence analysis

**Figure 3:**
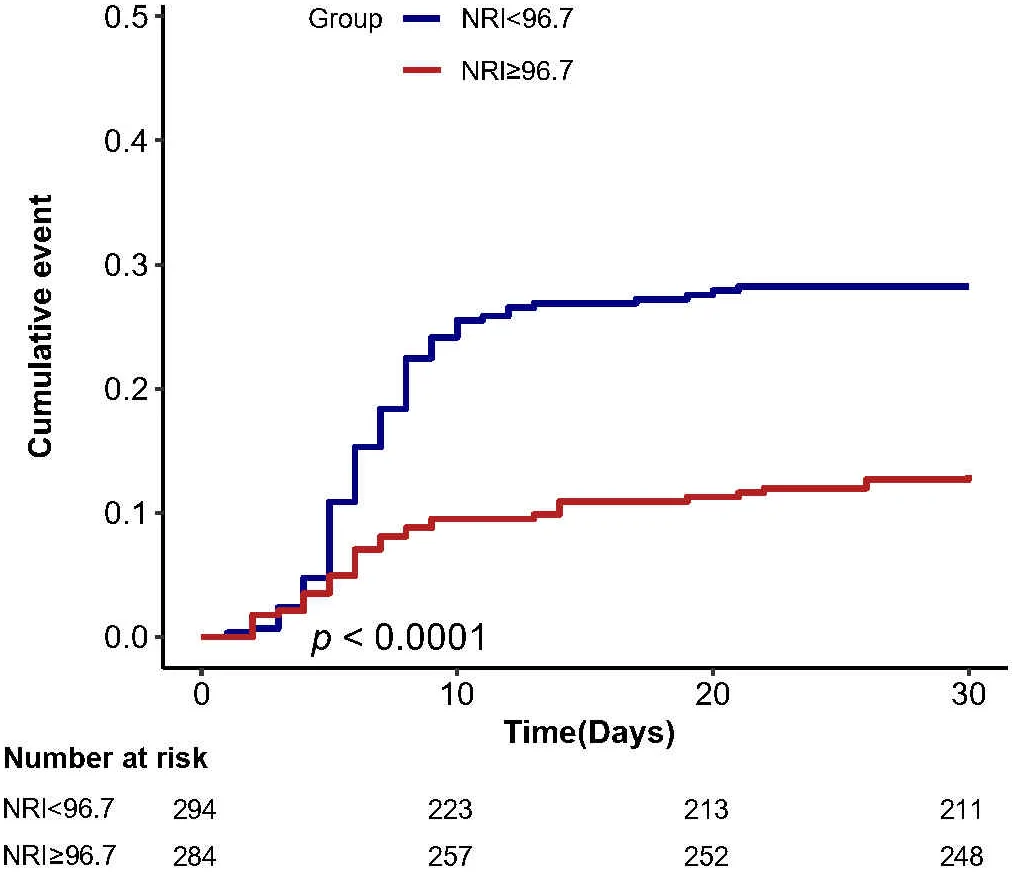
Cumulative incidence of 30-day postoperative complications.

The cumulative incidence of complications at 30 days postoperatively showed that patients in the high NRI group (NRI ≥ 96.7) had a lower cumulative risk of 30-day postoperative complications compared with the low NRI group (NRI < 96.7) (**Figure 3**).

## Discussion

### Key findings

A significant correlation was found between preoperative NRI levels and the incidence of 30-day postoperative complications in patients with gastric cancer. The incidence of 30-day postoperative complications in 578 gastric cancer surgery patients was 20.8%, and the incidence of postoperative complications was significantly higher in the low-NRI group than in the high-NRI group. After adjusting for confounders, the risk of 30-day postoperative complications was 2.89 times higher in the low-NRI group than in the high-NRI group. Apparently, preoperative NRI is an independent protective factor against 30-day postoperative complications in patients with gastric cancer.

### Relation to previous research

Nutritional conditions have been recognized as an important factor affecting the prognoses of cancer patients, attracting considerable interest (16–18). Malnutrition is common in patients with gastric cancer, having prevalence rates ranging from approximately 29.1% to 80.4% (19–21), and is associated with poor outcomes (16–18). In some studies, NRI as an indicator of nutritional assessment is mostly used preoperatively for the prediction of the postoperative outcomes of related diseases (22, 23). Notably, it is implemented using serum albumin and weight to assess the nutritional status of patients and can be independent of subjective factors. NRI demonstrates strong prognostic value in various populations, such as in cardiac surgery (12), breast cancer (13), or head and neck cancer (24, 25). Its clinical outcomes in patients with gastric cancer are inconsistent. Kim et al. assessed preoperative NRI scores in 958 stage II and III gastric cancer and found that patients with higher NRI scores significantly outperformed those with lower scores in terms of survival (26). Song et al. (27)specifically investigated the prognostic ability of NRI in stage III gastric cancer patients, demonstrating that NRI had prognostic significance with an optimal cutoff value of 99, correlating with short survival times. Similar conclusions were drawn from other studies on gastric cancer (28–30). However, these studies examined patient survival and did not explore postoperative complications. A previous study (31) focused only on the relationship between low preoperative NRI levels and wound complications after gastrectomy (NRI≥97.5 vs. NRI<97.5: OR=0.653, 95% CI:0.326–0.974, *P*=0.014). Furthermore, Choi et al. (32)found through meta-analysis that those with gastric cancer identified as malnourished (NRI<97.5) had a higher risk of postoperative wound complications compared to those with good nutritional status (NRI≥97.5). However, these studies did not examine other common postoperative complications, such as pneumonia, pulmonary embolism, and heart failure. In this study, we assessed 30-day postoperative complications in patients with gastric cancer by adjusting for confounders, revealing a strong association between NRI and postoperative complications. The results in our subgroup analysis were consistent with the primary results, suggesting that the results of the independent effect of NRI on 30-day postoperative complications in patients with gastric cancer have high reliability and clinical applicability. Cumulative incidence analysis showed that patients with low NRI had a higher risk of postoperative complications compared with the high NRI group. In addition, we found no deaths (Complications: Grade V) within 30 days postoperatively in both the low and high NRI groups. This may be attributed to the relatively short duration of the study, the optimization of clinical management, and the limitations of the sample size. Previous study (27) suggest that the low NRI group may present a higher risk of death at longer follow-up (1, 3 and 5 years).

The NRI, a quantitative laboratory marker, integrates serum albumin levels and body weight, which reflect the nutritional status and degree of immune suppression in patients with gastric cancer (33, 34). Serum albumin is not only an indicator of nutritional status but is also strongly associated with systemic inflammatory status (35). Inflammatory factors released by tumors may affect liver function, inhibit albumin synthesis, and even lead to the structural degeneration of albumin, considerably reducing serum albumin levels (36, 37). In addition, body weight is an important indicator of nutritional status, which directly affects the tolerance of patients with gastric cancer patients (38). Body weight plays an independent predictive role in the prognoses of patients with gastric cancer (39, 40). A low NRI score implies malnutrition and immunosuppression in patients, thus leading to a low tolerance for surgery or chemotherapy and predisposed to poor clinical outcomes. In summary, malnutrition and immunosuppression are the likely main factors affecting poor clinical outcomes in patients with gastric cancer. In addition to these factors, NRI assessment may involve other unknown mechanisms affecting prognosis, which need to be explained by further in-depth studies.

### Impact on clinical practice

The main significance of this study is that our findings suggest that NRI is strongly associated with 30-day postoperative complications in patients undergoing gastric cancer surgery. These findings will prompt healthcare professionals to identify high-risk patients and aid in the development of a preoperative nutritional management plan to improve the nutritional status of preoperative patients with gastric cancer and provide effective nutritional interventions. The aim was to reduce the rate of postoperative complications. In addition, our findings may contribute to future clinical studies of individualized nutritional control in gastric cancer patients to test whether the preoperative optimization of NRI can improve patients’ prognosis and adverse outcomes.

### Limitations

Our study has several limitations. First, it is a retrospective study, and despite our efforts to control potential confounding factors, we were unable to assess certain covariates that may have influenced the results. Second, the study only investigated the impact of NRI on early postoperative complications in patients with gastric cancer, without evaluating long-term postoperative outcomes. Third, it failed to assess the changes in NRI values after preoperative nutritional interventions and the possible impact on the cumulative incidence of postoperative complications.Despite adjusting for preoperative nutritional intervention status in multivariate models, the lack of standardized data on preoperative nutritional support limited our ability to assess its causal role. Future intervention studies should prioritize this variable to elucidate potential treatment effects. Lastly, we only collected clinical data from a single hospital, and thus the generalizability of the study findings may be limited. Future multicenter prospective studies are necessary to confirm our findings.

## Conclusion

Our study demonstrates that NRI plays a critical role in predicting adverse outcomes in gastric cancer patients, and that preoperative NRI levels correlate with the incidence of 30-day postoperative complications in patients with gastric cancer. These findings provide valuable guidance for clinical providers and emphasize the importance of accurately assessing and effectively managing populations with poor nutritional status.

## Data Availability

The original contributions presented in the study are included in the article/**Supplementary Material**. Further inquiries can be directed to the corresponding author.
