# Correction: Effect of preoperative nutritional risk index on 30-day postoperative complications in patients with gastric cancer: a retrospective cohort study

**DOI:** 10.3389/fonc.2026.1983817

**Published:** 2026-09-28

**Authors:** Yingfeng Zou, Ling Li, Kui Jia, Lei Tian, Minying He, Debin Huang

**Affiliations:** 1 Department of Gastrointestinal Surgery, The First Affiliated Hospital of Guangxi Medical University, Nanning, China; 2 Department of Nursing, The First Affiliated Hospital of Guangxi Medical University, Nanning, China

**Keywords:** gastric cancer, nutritional risk index, postoperative complication, surgery, cancer

There was a mistake in **Figure 1** as published. In the patient enrollment/flow diagram, the cutoff value for the NRI grouping was incorrectly displayed as “97.6”. The correct cutoff value is “96.7”, which is consistent with the ROC curve analysis described in the **Methods** section and the grouping criteria presented in **Table 1** of the original article. The corrected **Figure 1** appears below.

**Figure 1:**
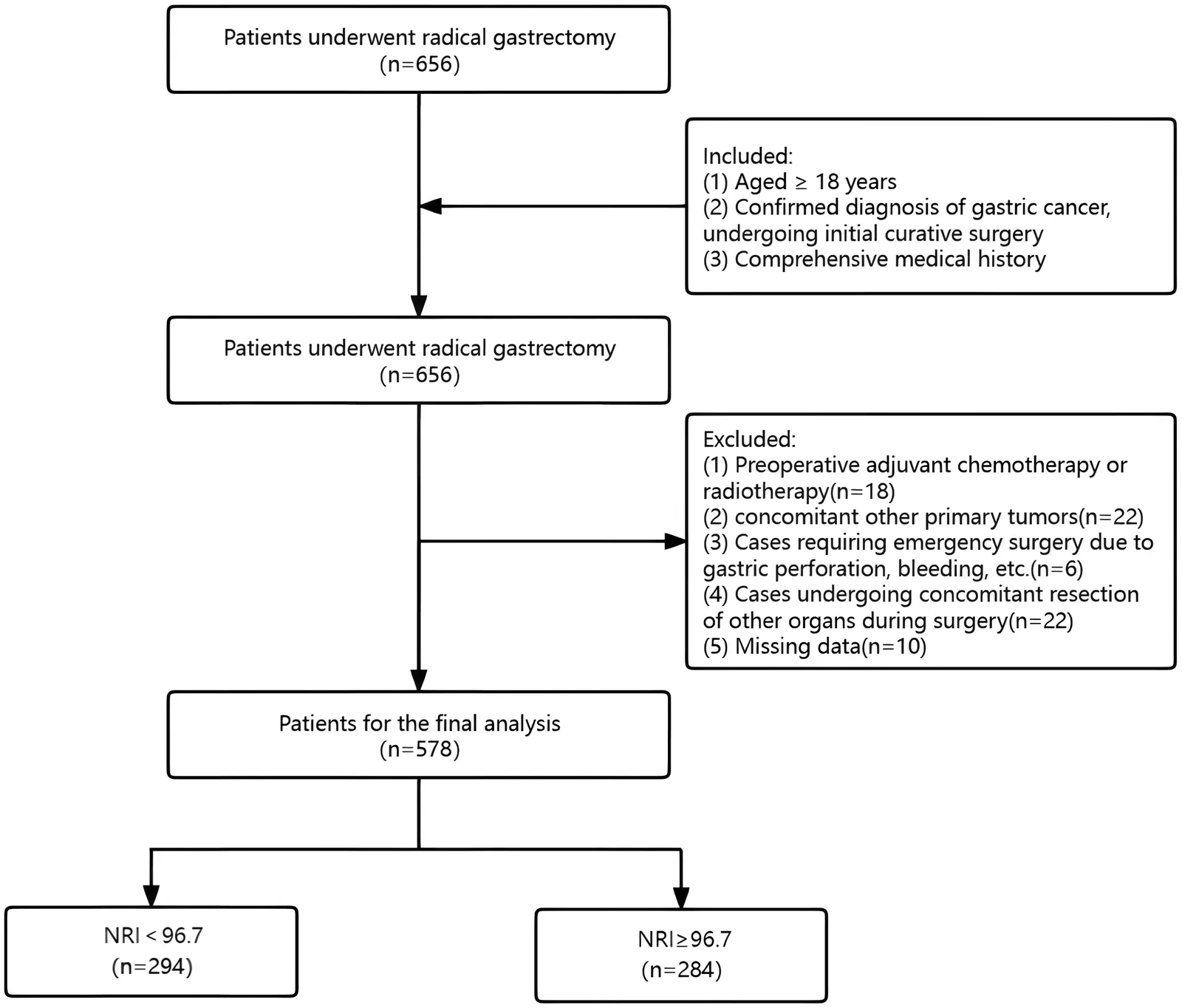
Flowchart of the study population. NRI, nutritional risk index.

The original version of this article has been updated.

