# Supplementary material for: Effect of preoperative nutritional risk index on 30-day postoperative complications in patients with gastric cancer: a retrospective cohort study
Source: Front Oncol. 2025 Jun 16;15:1475381. doi: 10.3389/fonc.2025.1475381 (PMC12206757; doi:10.3389/fonc.2025.1475381)
Supplement: Supplementary file 1 [file Image1.pdf]

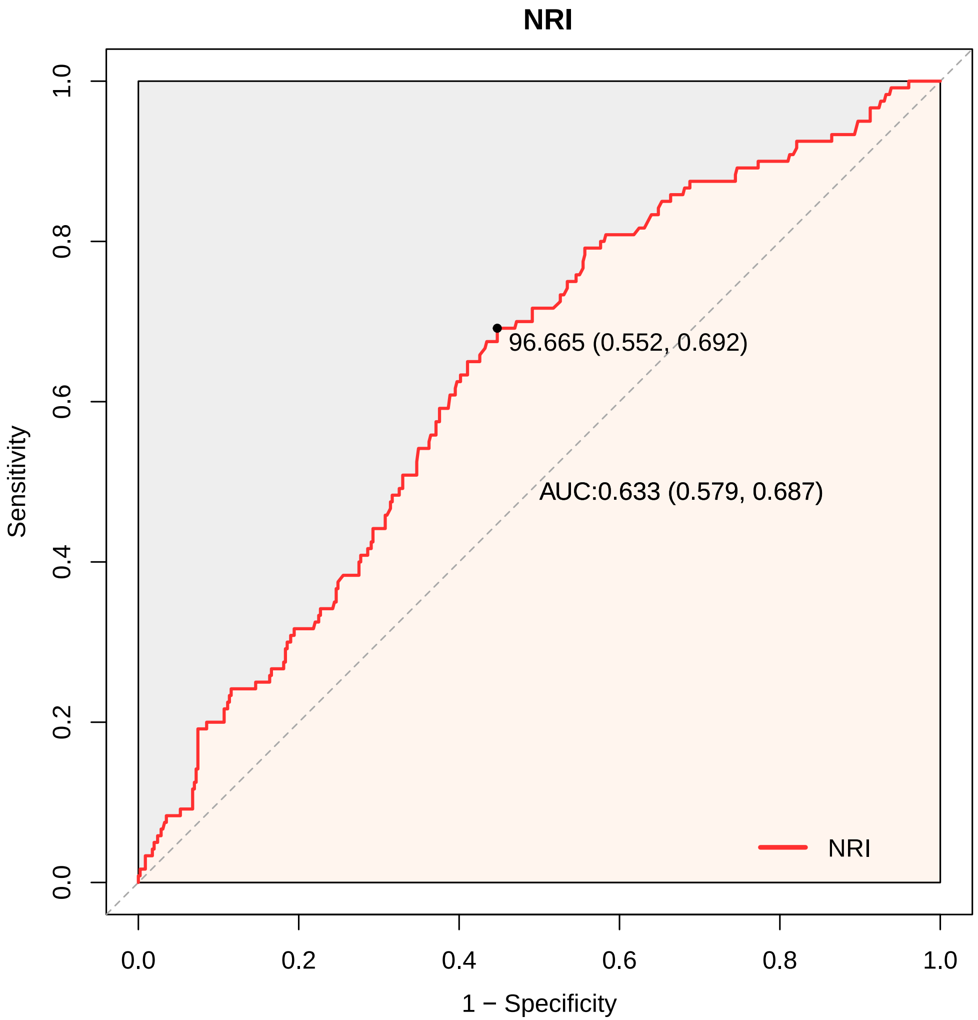

Variable	AUROC	Threshold	Specificity	Sensitivity	Accuracy
NRI	0.633	96.665	0.552	0.692	0.581

Figure S1. ROC curve of NRI as a predictor of postoperative complications
