# Supplementary material for: Effect of preoperative nutritional risk index on 30-day postoperative complications in patients with gastric cancer: a retrospective cohort study
Source: Front Oncol. 2025 Jun 16;15:1475381. doi: 10.3389/fonc.2025.1475381 (PMC12206757; doi:10.3389/fonc.2025.1475381)
Supplement: Supplementary file 2 [file Table1.pdf]

Supplementary Table 1. Type and number of 30-day postoperative complications in patients with gastric cancer

Types of complications	n（%）
pulmonary infection	84（70）
gastrointestinal fistula	10（8.3）
gastric atony	4（3.3）
pleural effusion	4（3.3）
others	18（15）

Supplementary Table 2. Baseline Characteristics of the Study Population According to Postoperative Complication

Variables	Total (n=578)	N-Complication (n=458)	Complication (n=120)	P value
Age, years	60.0 (52.0, 64.0)	58.0 (50.0, 63.0)	62.0 (57.0, 66.0)	<0.001
Male	361 (62.5)	274 (59.8)	87 (72.5)	0.011
BMI, kg/m2	21.8 (20.0, 23.8)	21.8 (20.0, 23.8)	21.9 (20.0, 23.9)	0.615
Smoking, n(%)	217 (37.5)	165 (36.0)	52 (43.3)	0.141
Drinking, n(%)	210 (36.3)	158 (34.5)	52 (43.3)	0.073
Preoperative nutritional intervention, n(%)				0.004
No	140 (24.2)	123 (26.9)	17 (14.2)	
Yes	438 (75.8)	235 (73.1)	103 (85.8)	
Gastrectomy, n(%)				<0.001
Proximal	15 (2.6)	7 (1.5)	8 (6.7)	
Distal	496 (85.8)	406 (88.6)	90 (75.0)	
Total	67 (11.6)	45 (9.8)	22 (18.3)	
Surgical approach,n(%)				<0.001
Open	105 (18.2)	60 (13.1)	45 (37.5)	
Laparoscopic	473 (81.8)	398 (86.9)	75 (62.5)	
ASA Class, n(%)				0.742
I	14 (2.4)	12 (2.6)	2 (1.7)	
II	446 (77.2)	355 (77.5)	91 (75.8)	
III	112 (19.4)	87 (19.0)	25 (20.8)	
IV	6 (1.0)	4 (0.9)	2 (1.7)	
TNM staging, n(%)				0.209
I	198 (34.3)	165 (36.0)	33 (27.5)	
II	135 (23.4)	105 (22.9)	30 (25.0)	
III	245 (42.4)	188 (41.0)	57 (47.5)	
Tumor differentiatio, n(%)				0.429
Un-classified	3 (0.5)	2 (0.4)	1 (0.8)	
Well	434 (75.1)	348 (76.0)	86 (71.7)	
Moderate	116 (20.1)	87 (19.0)	29 (24.2)	
Poor	25 (4.3)	21 (4.6)	4 (3.3)	
Hypertension, n (%)	115 (19.9)	89 (19.4)	26 (21.7)	0.585
Diabetes, n (%)	12 (2.1)	9 (2.0)	3 (2.5)	0.720
Coronary heart disease, n (%)	5 (0.9)	3 (0.7)	2 (1.7)	0.278
Cerebral Infarction, n (%)	5 (0.9)	4 (0.9)	1 (0.8)	1.000
Intraoperative blood transfusion, n(%)	129 (22.3)	88 (19.2)	41 (34.2)	<0.001
NRI	96.7 (91.1, 100.8)	97.5 (91.5, 101.5)	94.1 (88.9, 98.1)	<0.001
Albumin, g/L	37.4 (34.8, 40.1)	37.7 (35.5, 40.4)	35.2 (32.8, 38.3)	<0.001
Hemoglobin, g/L	124.0(108.7, 134.0)	124.4(110.8, 134.5)	123.0(93.5, 131.9)	0.066
Prealbumin, mg/L	215.3(177.6, 251.8)	218.2(182.1, 255.0)	200.8(165.2, 232.8)	<0.001
C-Reactive Protein, mg/L	8.0 (8.0, 32.3)	8.0 (8.0, 32.3)	8.0 (8.0, 32.3)	0.234
WBC, 109/L	6.1 (5.0, 7.1)	6.0 (4.9, 7.1)	6.2 (5.2, 7.2)	0.281
CA199, U/ml	6.3 (2.5, 17.2)	6.3 (2.5, 17.0)	6.1 (2.6, 17.6)	0.876
AFP, ng/ml	2.4 (1.8, 3.4)	2.4 (1.8, 3.4)	2.4 (1.8, 3.2)	0.931
Operation time, h	4.9 (4.2, 5.6)	4.9 (4.2, 5.5)	5.4 (4.4, 6.1)	<0.001
Intraoperative blood loss, ml	100 (80, 200)	100 (50, 200)	200 (100, 300)	<0.001

Supplementary Table 3. Univariate Analysis of the Factors Affecting 30-day Postoperative Complications

Variables	HR(95%CI)	P(Wald's test)
Gender: ref=male	0.59 (0.40,0.88)	0.010
Age	1.05 (1.03,1.07)	<0.001
BMI	1.02 (0.96,1.08)	0.597
NRI	0.95 (0.93,0.97)	<0.001
Smoking: ref=none	1.31 (0.92,1.88)	0.139
Drinking: ref=none	1.39 (0.97,2.00)	0.072
Hypertension: ref=none	1.12 (0.72,1.73)	0.614
Diabetes: ref=none	1.25 (0.4,3.93)	0.705
Coronary heart disease: ref=none	2.34 (0.58,9.47)	0.233
Cerebral Infarction:ref=none	1.00 (0.14,7.20)	0.995
Albumin	0.88 (0.85,0.92)	<0.001
Hemoglobin	0.99 (0.98,1.00)	0.013
C-Reactive Protein	1.00 (0.99,1.01)	0.397
Prealbumin	0.99 (0.99,1.00)	<0.001
WBC	1.06 (0.97,1.17)	0.185
CA199	1.00 (0.99,1.00)	0.967
AFP	0.99 (0.99,1.00)	0.618
Gastrectomy: ref= proximal		
distal	0.25 (0.12,0.52)	<0.001
Total	0.50 (0.22,1.12)	0.094
ASA Class: ref= I		
II	1.49 (0.37,6.05)	0.577
III	1.60 (0.38,6.77)	0.520
IV	2.60 (0.37,18.44)	0.340
TNM staging: ref= I		
II	1.42 (0.87,2.33)	0.164
III	1.46 (0.95,2.24)	0.083
Tumor differentiatio: ref= Un-classified		
Well	0.54 (0.08,3.87)	0.538
Moderate	0.69 (0.09,5.07)	0.716
Poor	0.44 (0.05,3.95)	0.464
Preoperative nutritional intervention, ref=none	2.22 (1.28,3.87)	0.005
Intraoperative blood loss	1.01 (1.00,1.02)	<0.001
Operation time	1.44 (1.25,1.65)	<0.001
Surgical approach: ref= Open	0.31 (0.21,0.45)	<0.001
Intraoperative blood transfusion: ref=none	1.97 (1.35,2.88)	<0.001

NRI, nutritional risk index; BMI, body mass index; ASA, American Society of Anesthesiologists; TNM, Tumor-Node-Metastasis; WBC, white blood cell; AFP, Alpha-Fetoprotein.
